# From Blood Count Parameters to ROP Risk: Early Hematological Predictors in Preterm Infants

**DOI:** 10.3390/medicina61091581

**Published:** 2025-09-01

**Authors:** Laura Bujoreanu Bezman, Carmen Tiutiuca, Florin Ciprian Bujoreanu, Nicoleta Cârneciu, Mihaela Crăescu, Florentin Dimofte, Elena Niculeț, Aurel Nechita

**Affiliations:** 1 Department of Ophthalmology, “Sf. Ioan” Emergency Clinical Hospital for Children, 800487 Galati, Romania; laura.bezman@ugal.ro; 2 Faculty of Medicine and Pharmacy, “Dunarea de Jos” University of Galati, 800385 Galati, Romania; nicoleta.carneciu@ugal.ro (N.C.); mihaela.craescu@ugal.ro (M.C.); florentin.dimofte@ugal.ro (F.D.); elena.niculet@ugal.ro (E.N.); aurel.nechita@ugal.ro (A.N.); 3 Department of Ophthalmology, “Sf. Apostol Andrei” Emergency Clinical Hospital, 800578 Galati, Romania

**Keywords:** ROP, biomarkers, hematological predictors, anemia, thrombocytopenia, risk factors, blood transfusions

## Abstract

*Background and Objectives*: Retinopathy of prematurity (ROP) persists as a major global cause of preventable childhood blindness. While early diagnosis and timely intervention can significantly mitigate visual loss, research is increasingly focused on identifying novel prognostic factors, with hematological markers emerging as a promising avenue for refining ROP risk prediction. This study aimed to assess the association of hemoglobin levels, red blood cell count, platelet count, and blood transfusions with the risk of developing ROP. *Materials and Methods*: We conducted a retrospective study involving 140 preterm infants (gestational age ≤ 34 weeks) admitted to a neonatal intensive care unit between 2021 and 2024. Hematological parameters were monitored sequentially during the first 28 days of life, and ROP screening was performed in accordance with international guidelines. Statistical analyses evaluated associations between hematological markers and the risk of developing ROP. *Results*: Anemia prevalence was significantly higher in infants who developed ROP (83.1%) compared with those who did not (60.3%), conferring an increased risk of ROP (OR = 3.239; *p* = 0.001). Red blood cell transfusions were linked to a higher likelihood of developing ROP (OR = 3.088; *p* = 0.001), while platelet transfusions showed a similar association (OR = 2.807; *p* = 0.027). Platelet counts were significantly lower on days 7, 14, and 21 in the ROP group, and thrombocytopenia was associated with an elevated risk of disease (OR = 3.542; *p* = 0.001). *Conclusions*: Early hematological imbalances (anemia, thrombocytopenia) and the requirement for blood product transfusions are significantly associated with an increased risk of ROP. Integrating the monitoring of these specific parameters into existing ROP screening protocols could enhance early identification of vulnerable preterm infants, enabling more targeted surveillance and potential preventative strategies.

## 1. Introduction

Retinopathy of prematurity (ROP) remains a leading preventable cause of childhood blindness globally. This condition, specific to preterm infants, is characterized by disordered retinal vascular development. While early detection and appropriate treatment yield favorable visual outcomes, progression without timely intervention can lead to severe disease with a significant risk of irreversible visual impairment or blindness [1]. Consequently, the early identification of high-risk preterm infants and the timely initiation of treatment when indicated represent critical challenges in ROP management [2].

Despite significant advances in neonatal care, ROP remains a major threat to vision in preterm infants, partly due to an incomplete understanding of its underlying mechanisms [3]. Current models describe ROP progression as occurring through two sequential, pathophysiologically distinct phases. The initial hyperoxic phase, which typically occurs between 22 and 30 weeks of postmenstrual age (PMA), involves postnatal exposure to relatively high oxygen levels, leading to vaso-obliteration and arrested physiological retinal vascular development [4]. Subsequently, the hypoxic phase emerges between 31- and 36-weeks PMA, characterized by pathological neovascularization driven by Vascular Endothelial Growth Factor (VEGF) and other pro-angiogenic cytokines in response to retinal hypoxia [5]. Hypoxia-inducible factors (HIFs) play a central role in this process, regulating the transcription of VEGF and other mediators involved in retinal neovascularization [6]. A critical disruption in the dynamic equilibrium between retinal oxygen supply and the metabolic demands of the developing retina initiates and sustains these pathological cascades. Oxygen supplementation, although essential in neonatal intensive care, remains one of the most debated modifiable factors in ROP, as both hyperoxia and intermittent hypoxia contribute to retinal vascular instability and disease progression [7]. Consequently, targeted management of factors influencing oxygen delivery during both phases represents a key strategy for reducing ROP risk and progression [8]. Beyond oxygen imbalance, nutrition also influences these pathways. Early enteral feeding, particularly with fortified human milk, increases circulating Insulin-like Growth Factor 1 (IGF-1) levels, a growth factor essential for normal retinal vascular development. Low IGF-1 levels, typical of preterm infants, have been consistently associated with impaired vascular maturation and higher risk of severe ROP [9].

Within this pathophysiological framework, recent research has increasingly focused on identifying hematological biomarkers predictive of ROP risk. Erythrocyte-related parameters, such as hemoglobin concentration and red blood cell (RBC) count, have emerged as key candidates, given their critical function in oxygen transport and tissue oxygen homeostasis [10,11]. These hematological indicators may serve as indirect proxies for retinal oxygen availability and exert significant influence over physiological and pathological vascular development pathways [12].

Anemia in preterm infants, characterized by impaired erythropoiesis, represents a significant risk factor for ROP. This association is complex, involving both the direct consequences of reduced hemoglobin levels and the potential impacts of therapeutic interventions, particularly RBC transfusions [13]. Hemoglobin critically modulates retinal vascularization by determining oxygen delivery capacity. Disruption of the delicate equilibrium between retinal metabolic demand and oxygen supply can thereby promote pathological vascular development pathways [14].

Multiple recent studies have established RBC transfusions as modifiable risk factors in the pathogenesis of ROP. While essential for managing anemia in the neonatal intensive care unit (NICU), these transfusions induce a rapid shift from endogenous fetal hemoglobin (HbF) to donor-derived adult hemoglobin (HbA). Critically, HbA exhibits a lower oxygen affinity than HbF, promoting a rightward shift of the oxygen dissociation curve. This reduces oxygen unloading capacity within the retinal microvasculature and exacerbates local oxidative stress due to the distinct biochemical properties of HbA [15,16]. Furthermore, both the timing and cumulative frequency of transfusions correlate with increased ROP risk, suggesting a potential dose-dependent effect on disease progression [17].

While current evidence linking thrombocytopenia to ROP remains limited, accumulating studies are now elucidating this potential association. Although few studies have directly assessed the influence of low platelet counts on ROP pathogenesis, scientific interest in this area is expanding [18,19]. The predominant hypothesis posits that platelets actively participate in regulating pro-angiogenic signaling, particularly the bioavailability of VEGF-A, a central mediator of retinal vascularization. Suboptimal platelet levels may impair the targeted delivery of these factors to the developing retina, potentially disrupting physiological vascular patterning. Consequently, vigilant monitoring and proactive management of thrombocytopenia may offer viable strategies for preventing or mitigating ROP, particularly in high-risk neonates [20,21].

Given the intricate pathophysiology of ROP and the significant influence of hematological imbalances on retinal vascular development, this study aims to investigate the association between specific erythrocyte and platelet parameters, as well as transfusion practices, since these are routine and easily measurable markers increasingly recognized as potential predictors of ROP [11]. The primary objective is to identify predictive hematological biomarkers suitable for incorporation into neonatal screening protocols, enabling enhanced risk stratification and improved preventive strategies for high-risk preterm infants.

## 2. Materials and Methods

Design: This retrospective study was conducted in the Neonatal Intensive Care Units of the “Sf. Apostol Andrei” Emergency Clinical Hospital and the “Sf. Ioan” Emergency Clinical Hospital for Children, Galati, Romania, and included 140 preterm infants born between January 2021 and December 2024. Of these, 77 preterm infants (55.0%) were diagnosed with ROP, while 63 (45.0%) had no clinical signs of the disease.

Inclusion criteria:

This study included preterm infants who met all of the following conditions: gestational age ≤ 34 weeks, birth weight ≤ 2000 g, survival for at least 7 days, availability of a sufficient number of hematological tests performed during hospitalization, and completion of ophthalmologic screening for ROP.

Exclusion criteria:

The exclusion criteria for this study target patients with major congenital malformations, severe hematological or infectious conditions that could influence biological parameters, as well as patients with incomplete essential medical data—particularly regarding biological monitoring and ophthalmologic evaluations.

Data collection and variables analyzed:

The information was collected retrospectively from clinical observation charts, laboratory registers, and ophthalmologic reports during the hospitalization period. Eventually, the data were compiled into an anonymized electronic database, with each patient identified by a unique numeric code to ensure confidentiality.

The following categories of variables were processed in each case:

- Demographic data: gestational age in weeks (GA), birth weight in grams (BW), and sex;
- Hematological parameters: hemoglobin level (Hb), RBC count, and platelet count (PLT), collected sequentially on days 1, 3, 5, 7, 14, 21, and 28 of life, depending on the length of hospitalization;
- Transfusions: type of product administered (RBC or PLT concentrate) and number of units transfused;
- Ophthalmologic findings: the presence or absence of ROP, the highest stage observed, the location of retinal changes according to the International Classification of Retinopathy of Prematurity (ICROP), and any possible therapeutic indications.

Hematological assessment: Biological tests were performed sequentially, in accordance with institutional protocols, on days 1, 3, 5, 7, 14, 21, and 28 of life. Given the variable duration of hospitalization, not all patients underwent complete monitoring throughout the entire period. The distribution was as follows: 111 preterm infants were monitored up to and including day 28, 13 up to day 21, 7 up to day 14, and 9 only up to day 7. All laboratory tests were performed on automated equipment that was periodically calibrated, with no methodological changes during the study period.

Transfusion data: Each preterm infant included in the study was documented on any transfusion administered during the neonatal period, specifying the type of product transfused (RBC or PLT concentrate) and the total number of units received during hospitalization. RBC transfusions were primarily indicated for the treatment of neonatal anemia, in accordance with unit protocols. Similarly, PLT transfusions were administered in cases of significant thrombocytopenia or increased risk of bleeding, according to the guidelines in the neonatal intensive care unit.

Ophthalmologic screening: All patients in the study cohort were examined for ROP using indirect ophthalmoscopy with a 20D lens, after mydriasis was induced with 2.5% phenylephrine and 0.5% tropicamide. The first evaluation was performed either at 4 weeks of postnatal age or between 31 and 33 weeks of PMA, depending on eligibility. Retinal lesions were classified according to ICROP criteria, and the frequency of ophthalmologic follow-up examinations was adjusted based on the severity and location of the findings. Observation continued until complete retinal vascularization or full regression of the disease. Cases that met the criteria for Type 1 ROP were referred to specialized centers for treatment initiation in accordance with current national and international guidelines.

Statistics analysis: Data processing was performed using IBM SPSS Statistics version 29.0. Quantitative variables were presented as average ± standard deviation or as median and interquartile range, depending on their distribution. To compare hematological parameters between the groups with and without ROP, the T-Student test was used for normally distributed data, and the Mann–Whitney U test for nonparametric distributions. Comparisons involving more than two groups or multiple time points were conducted using ANOVA for parametric variables or the Kruskal–Wallis test for nonparametric data. Associations between class variables were analyzed using Pearson’s Chi-square test, and Fisher’s exact test was applied when expected frequencies were low. The risk of ROP occurrence in relation to factors such as anemia, thrombocytopenia, or transfusions was estimated by calculating odds ratio (OR), with a 95% confidence interval (CI95%). A *p*-value of less than 0.05 was considered statistically significant.

Ethical approval: This study was conducted in accordance with the ethical principles outlined in the Declaration of Helsinki and received approval from the ethics committees of the following institutions: “Sf. Apostol Andrei” Emergency Clinical Hospital, Galati, Romania (approval no. 2955, 6 February 2024), “Sf. Ioan” Emergency Clinical Hospital for Children, Galati, Romania (approval no. 2844, 29 February 2024), the Ethical Committee of the Medical College of Galati (approval no. 138, 9 February 2024), and “Dunărea de Jos” University of Galati, Romania (approval no. 7284, 19 March 2024). Written informed consent was obtained from all participants prior to inclusion in the study. All data were fully anonymized before statistical processing to ensure the protection of patient identity throughout all stages of analysis.

## 3. Results

### 3.1. General Characteristics of the Study Population

**Figure 1:**
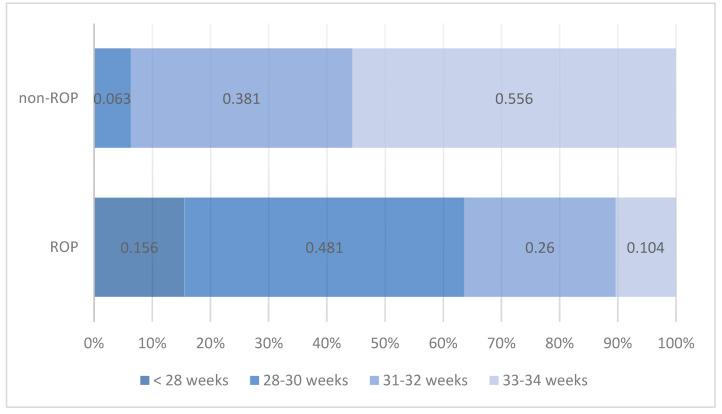
Distribution of preterm infants by gestational age and presence of ROP.

This study included 140 preterm infants with a GA of ≤34 weeks. Among them, 55.0% were diagnosed with ROP, while 45.0% showed no signs of the disease (non-ROP). The distribution according to the GA revealed that 8.6% of the infants (*n* = 12) were born at less than 28 weeks, 29.3% (*n* = 41) were between 28 and 30 weeks, 31.4% (*n* = 44) were between 31 and 32 weeks, and 30.7% (*n* = 43) had a GA between 33 and 34 weeks. Statistical analysis revealed significant differences in GA between preterm infants with and without ROP. All infants with a GA of less than 28 weeks developed ROP, and the same trend was predominantly observed among those with a GA between 28 and 30 weeks. Among all patients diagnosed with ROP, 63.7% had a GA of 30 weeks or less. In contrast, in the non-ROP group, none of the infants had a GA under 28 weeks, and only 6.3% had a GA between 28 and 30 weeks (Table 1, Figure 1).

### 3.2. Erythrocyte Profile and the Role of RBC Transfusions in the Development of ROP

**Figure 2:**
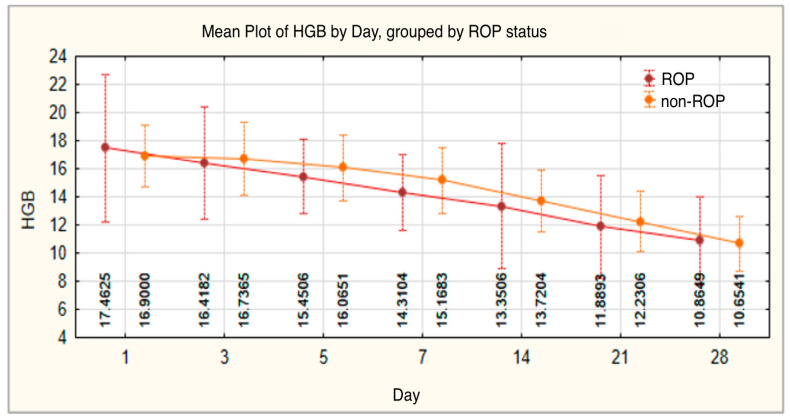
Hemoglobin—evolution of values in the ROP group and the non-ROP group.

Hemoglobin levels showed a progressive decrease throughout the monitoring period, from an initial average of 17.2094 ± 4.13786 to 10.7946 ± 2.77842 on day 28 of life. On day 7 of life, hemoglobin levels were significantly higher in infants without ROP (*p* = 0.048), and by the end of the monitoring period, values were similar between the two groups (Table 2, Figure 2).

**Figure 3:**
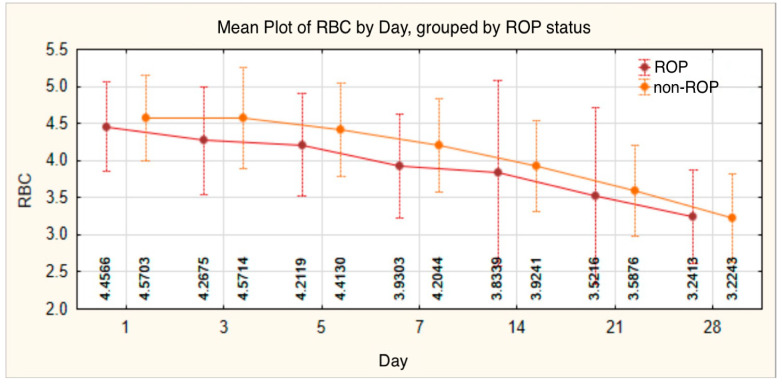
Red blood cell count—evolution of values in the ROP group and the non-ROP group.

RBC values also followed a downward trend throughout the monitoring period, decreasing from an initial mean of 4.5078 ± 0.59300 on day 1 to 3.2355 ± 0.62008 by the end of the observation period. In the ROP group, values remained lower during the monitoring period, with statistically significant differences on day 3 (4.2675 ± 0.72217, *p* = 0.012) and day 7 (3.9303 ± 0.69662, *p* = 0.016) compared with the non-ROP group (Table 3, Figure 3).

**Figure 4:**
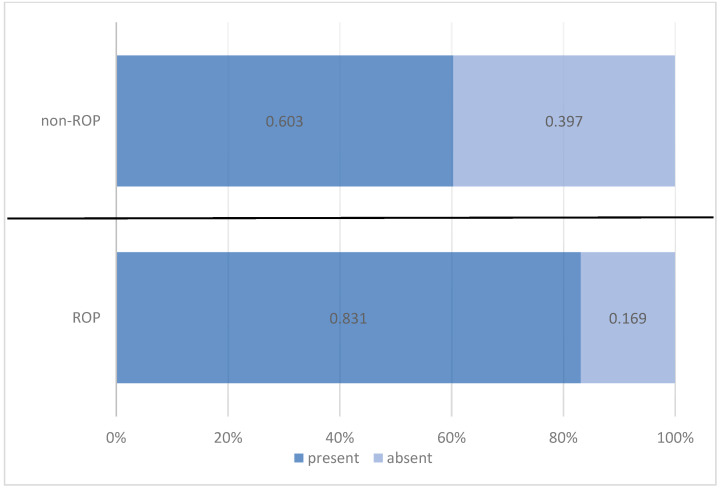
Comparison of preterm infants by anemia and ROP status.

Anemia was recurrent in approximately three-quarters of the preterm infants in the cohort (72.9%), with a higher prevalence among those with ROP (83.1%) compared with those without ROP (60.3%). This difference indicates a statistically significant association between anemia and the presence of ROP. Risk analysis showed that preterm infants with anemia had a 3.239-times higher likelihood of developing ROP compared with those without anemia (Table 4, Figure 4).

Across the entire cohort, anemia onset occurred between days 13 and 14 of life, with an average of 13.59 ± 7.606 and a median of 12.50, ranging from day 1 to day 28. In infants with ROP, anemia developed slightly earlier, on day 13 (13.03 ± 7.307), while in those without ROP, it appeared around days 14–15 (14.53 ± 8.097) (Table 5).

**Figure 5:**
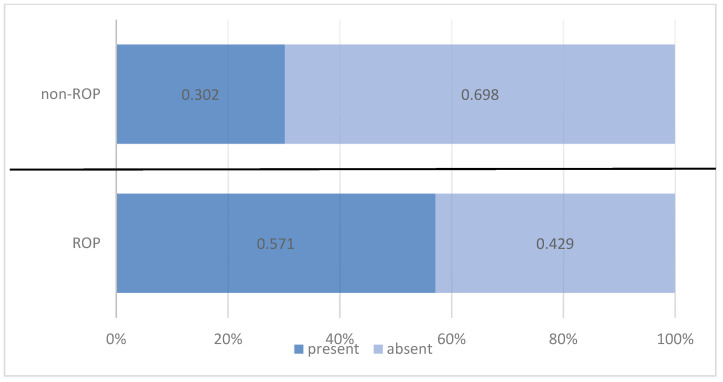
Comparison of preterm infants by RBC transfusion status and ROP status.

Nearly half of the preterm infants monitored (45.0%) required RBC transfusions; however, the administration of RBC transfusions was significantly more frequent among those with ROP (57.1%) as compared with the non-ROP group (30.2%). Risk analysis indicated that RBC transfusions were associated with a 3.088 times increased risk of developing ROP (Table 6, Figure 5).

### 3.3. Platelet Profile and the Role of Platelet Transfusions in the Development of ROP

**Figure 6:**
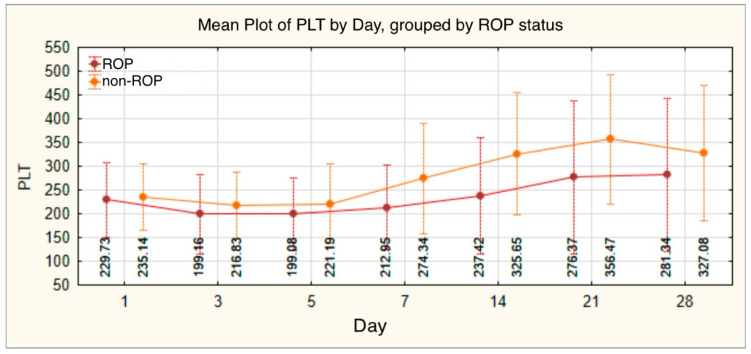
Platelet count—evolution of values in the ROP group and the non-ROP group.

Platelet levels showed variations throughout the monitoring period, starting with an initial average value of 232.16 ± 75.325, followed by successive fluctuations and a general upward trend, reaching a final mean of 296.59 ± 156.638. Significant differences, with lower values in the ROP group, were observed on day 7 (ROP: 212.95 ± 88.906 vs. non-ROP: 274.34 ± 115.747, *p* = 0.001), day 14 (*p* < 0.001), and day 21 (*p* = 0.005) (Table 7, Figure 6).

**Figure 7:**
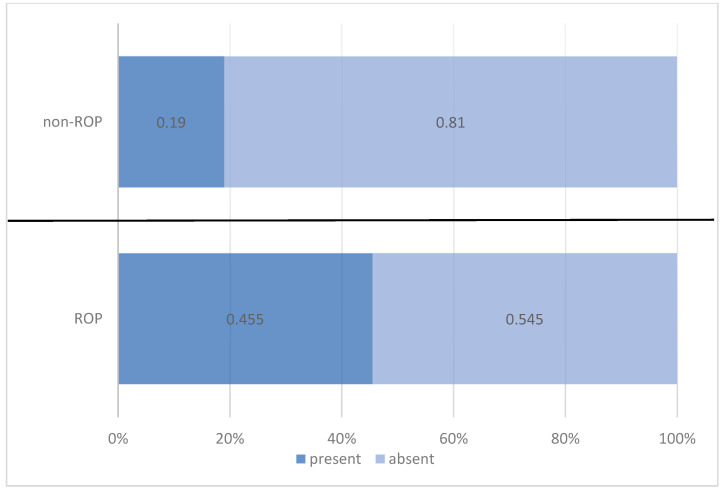
Comparison of preterm infants by thrombocytopenia and ROP status.

Thrombocytopenia occurred in one-third of the preterm infants (33.6%), with a statistically significantly higher percentage among those diagnosed with ROP (45.5%) compared with only 19% of those without ROP. Risk analysis indicated that preterm infants with thrombocytopenia had a 3.542 times higher likelihood of developing ROP (Table 8, Figure 7).

Across the entire cohort, thrombocytopenia onset occurred around days 7–8 of life, with an average of 7.49 ± 5.830 and a median of 5.00, ranging from day 1 to day 21. In preterm infants diagnosed with ROP, thrombocytopenia developed slightly later, around day 8 (average 8.06 ± 6.053), while in those without ROP, it occurred earlier, around days 5–6 (mean 5.83 ± 4.988). However, the difference was not statistically significant (Table 9).

**Figure 8:**
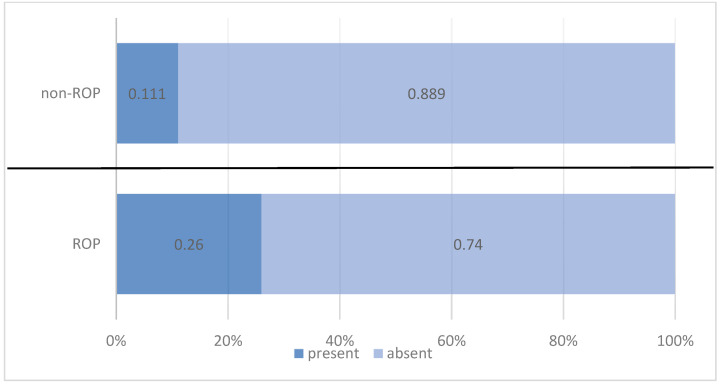
Comparison of preterm infants by PLT transfusion and ROP status.

Platelet transfusions were more frequently administered to preterm infants with ROP, being reported in 26.0% of these cases compared with only 11.1% of infants without ROP. The difference was statistically significant and indicated that platelet transfusions increased the risk of developing ROP by 2.807 times (Table 10, Figure 8).

## 4. Discussion

To prevent complications associated with ROP, new risk prediction algorithms are currently being developed, some of which also integrate serum biomarkers. Although promising, these algorithms have not yet been validated or widely accepted, so screening continues to rely on conventional clinical criteria, which are often insufficiently sensitive or specific [22]. Serum represents a promising source of measurable compounds, easily collected through minimally invasive methods, which makes it suitable for neonatal monitoring in the context of ROP risk [23]. Predictive models should integrate serum biomarkers with clinical data to improve case detection and reduce unnecessary ophthalmologic examinations among preterm infants at minimal risk [24].

Based on these considerations, our study focused on the progression of routine hematological parameters during the first 28 days of life, with the aim of assessing their potential as dynamic markers for predicting the risk of ROP. The longitudinal analysis of hemoglobin, red blood cells, and platelet values, correlated with the onset of anemia or thrombocytopenia as well as the need for transfusions, allowed us to highlight significant differences between preterm infants who developed ROP and those who did not. Given the progressive and staged nature of ROP pathogenesis, our sequential follow-up allowed us to capture differences that emerged only at specific time points, notably days 3, 7, 14, and 21, supporting the value of dynamic monitoring rather than relying on isolated time points. To our knowledge, no previous studies have monitored these parameters in such a detailed dynamic manner within the same cohort of preterm infants at risk for ROP.

Anemia in preterm infants is a complex condition with multisystemic impact, particularly affecting high-energy-demanding organs. The retina is one of the most hypoxia-sensitive tissues, and its elevated metabolic requirements during the neonatal period make it especially vulnerable in the context of prematurity [25]. Under these circumstances, anemia significantly contributes to the imbalance of retinal angiogenesis due to its reduced oxygen-carrying capacity, promoting hypoxia and triggering molecular mechanisms involved in the pathogenesis of retinopathy of prematurity [26,27]. Impaired hemoglobin synthesis, together with low absolute levels and sudden fluctuations, exacerbate the ischemic retinal environment, stimulating the activation of HIFs and, subsequently, the expression of proangiogenic mediators such as VEGF, directly involved in the development of pathological neovascularization [28]. These mechanisms highlight that HIF-mediated VEGF upregulation represents a major pathway linking hematological imbalance to abnormal retinal angiogenesis in ROP.

The dynamic assessment of hemoglobin levels, as performed in our study, has also been linked in previous research to more aggressive forms of the disease, highlighting the importance of continuous hematologic monitoring [29]. Physiologically, in term neonates, hemoglobin levels usually range between 14 and 24 g/dL at birth, decreasing to 12–20 g/dL during the first two weeks of life [30]. However, in preterm infants, values vary physiologically depending on the gestational age, the day of life, and the clinical context, which makes it difficult to define a single, universally applicable threshold for anemia. Since there is no widely standardized or accepted cut-off value in this regard, the present study used reference ranges adjusted according to the GA and postnatal evolution, based on intervals described in the existing literature [31]. During the first week of life, anemia was diagnosed at hemoglobin values between 13.5 and 12.5 g/dL, depending on the degree of prematurity. In the second week, thresholds ranged from 12.5 to 11.5 g/dL, while in the third and fourth weeks, values between 11.5 and 11 g/dL were used. These thresholds were subsequently applied in analyzing the timing of anemia onset within the study cohort.

Numerous recent studies have highlighted the role of low hemoglobin levels in the pathogenesis of ROP. Gudu et al. reported significantly lower average levels in the first 48 h among newborns who developed ROP (16.32 ± 3.05 g/dL) compared with those without ROP (17.82 ± 2.55 g/dL; *p* = 0.002) [10]. Similarly, Maeda et al. found that Hb < 9.9 g/dL on day 28 was correlated with treatment-requiring ROP [12]. In addition, Pai et al. reported that Hb < 8 g/dL during the third week of life was associated with advanced forms of ROP, whereas levels above 10 g/dL were linked to a more favorable clinical course [32].

In our study, differences between the groups became statistically significant on day 7, when preterm infants without ROP had an average hemoglobin level of 13.0152 ± 2.67082 g/dL, compared with 11.8485 ± 2.35464 g/dL in those with ROP (*p* = 0.048). In the third week of life (day 21), the mean hemoglobin values were 11.8893 ± 3.64244 g/dL in the ROP group and 12.2306 ± 2.12722 g/dL in the non-ROP group, while in the fourth week (day 28), the values were 10.8649 ± 3.12583 g/dL and 10.6541 ± 1.93500 g/dL, respectively. These findings indicate a continuous decline in hemoglobin levels in both groups; however, the decrease was more emphasized in preterm infants who developed ROP, supporting the hypothesis of increased hematological vulnerability in this population.

RBC is an essential parameter in evaluating hematopoietic maturity and tissue oxygenation capacity in newborns. Christensen et al. reported that RBC values are physiologically lower in preterm infants compared with term neonates [33,34]. In a study conducted on a cohort of low-birth-weight preterm infants, Ochiai et al. reported day 1 RBC values ranging between 3.90 and 6.10 × 10^12^/L in infants with a GA < 36 weeks [35]. In our study, the average RBC value on day 1 was 4.5078 ± 0.59300 × 10^12^/L, which is consistent with the international reference ranges available. Although no significant differences were observed on day 1 between preterm infants with and without ROP (4.4566 ± 0.6037 vs. 4.5703 ± 0.5782; *p* = 0.261), these differences became statistically significant starting on day 3 (4.2675 ± 0.72217 in the ROP group) and remained so on day 7 (3.9303 ± 0.69662 in the ROP group), suggesting that a reduced RBC count may serve as an early indicator of the risk of developing ROP.

The international literature provides relatively limited data that directly investigate RBC values in preterm infants with ROP, as most studies focus on derived parameters such as hemoglobin or hematocrit [36]. Fevereiro-Martins et al. found significantly lower values during the first week in ROP infants (median: 4.0 × 10^12^/L) compared with non-ROP (4.3 × 10^12^/L; *p* < 0.001), though this association lost significance in multivariate analysis due to GA and transfusion effects [37]. For comparison, in term neonates, the median RBC count is approximately 4.64 × 10^12^/L [38].

Teofili et al. conducted a retrospective analysis of a cohort of 100 preterm infants with a GA < 30 weeks, highlighting that RBC transfusions are associated with a significantly increased risk of severe ROP. The risk was more than six times higher in infants who received transfusions at an early stage of disease progression, and repeated administration was correlated with up to an eightfold increase in disease risk. The authors suggest that early transfusional exposure may contribute to retinal angiogenic imbalance by reducing fetal hemoglobin levels and inducing oxidative stress [39].

HbF plays a crucial role in maintaining efficient oxygenation in the immature retina due to its higher affinity for oxygen compared with HbA [40,41]. Maintaining elevated levels of HbF during the neonatal period contributes to a stable oxygen supply, limiting the activation of pathological proangiogenic pathways. In contrast, a sudden shift toward HbA, often triggered by transfusions, may disrupt the balance of retinal oxygenation and promote the development of ROP [10].

Several clinical studies have highlighted the association between blood transfusions and the risk of ROP. Slidsborg et al. identified transfusions, along with mechanical ventilation, as independent risk factors for the development of the disease [42]. Other studies suggest that maintaining a high level of HbF, including through the use of umbilical cord blood, may reduce the incidence of severe ROP by contributing to a more stable hematologic balance during this vulnerable period. As such, transfusion management plays a central role in disease prevention, and regulating the ratio between HbF and HbA is seen as a promising therapeutic direction [43].

Our findings support these observations, highlighting a significant association between RBC transfusion and the risk of ROP. Nearly half of the preterm infants (45.0%) required blood transfusions, with a significantly higher frequency among those with ROP (57.1%) compared with the non-ROP group (30.2%). Risk analysis indicated that preterm infants who received transfusions had a 3.088-times increased risk of developing ROP (OR = 3.088; 95% CI: 1.530–6.232; *p* = 0.001). These data position RBC transfusions not only as a marker of clinical severity but also as a potential direct contributor to retinal vascular imbalance.

In line with these results, Lust et al. found that transfusions administered in the first 10 days of life were associated with a 3.84-times increased risk of severe ROP, while Zhu et al., in a meta-analysis of over 15,000 preterm infants, reported an overall 1.50-times higher risk, rising to 1.77-times for GA ≤ 32 weeks [44,45].

These observations reinforce the hypothesis that RBC transfusions, especially when administered early and repeatedly, may contribute to retinal oxygenation instability and the progression of ROP. Therefore, they should not be viewed solely as hematologic support interventions, but also as factors requiring careful management within a balanced approach that weighs clinical necessity against potential risk [46].

Thrombocytopenia is defined as a platelet count < 150.000 × 10^3^/µL. Platelet values are physiologically higher in term neonates, with a median around 258 ×10^3^/µL (range 150–450 × 10^3^/µL) [38]. Although data in the literature remain limited, an increasing number of recent studies are investigating the involvement of this hematological disorder in the pathogenesis of ROP [47,48]. Platelets play an active role in the regulation of angiogenesis through their ability to store and release proangiogenic factors such as VEGF. Through this mechanism, they may contribute to maintaining functional vascular balance in the immature retina, supporting the hypothesis that a reduced platelet count may favor the onset and progression of severe forms of ROP [49]. This association is being investigated particularly in very low birth weight preterm infants, in whom aggressive forms of ROP are more frequently correlated with low platelet counts [50].

Moreover, in the context of hypoxia or excessive oxygen exposure, thrombocytopenia may exacerbate the imbalance of retinal angiogenesis. An increasing number of studies support the monitoring of platelet levels in preterm infants and the inclusion of this parameter in ROP screening and surveillance algorithms. In a meta-analysis that included 1762 preterm infants, of whom 747 were diagnosed with ROP, Yan-Hong et al.l observed a significant mean difference between the two groups (−18.65 × 10^9^/L; 95% CI: −22.80 to −14.50; *p* < 0.00001), thus supporting the role of thrombocytopenia as a potential risk marker and advocating for the inclusion of this parameter in neonatal evaluation [51].

Similar findings were reported by Nedime et al. who analyzed 137 preterm infants (GA ≤ 34 weeks) and found that first week thrombocytopenia correlated significantly with ROP, with lower mean platelet counts in affected infants (222 ± 69 × 10^3^/μL) and in those requiring treatment (214 ± 62 × 10^3^/μL) compared with those without ROP (280 ± 103 × 10^3^/μL; *p* = 0.002) [52]. In our cohort, the onset of thrombocytopenia in the ROP group occurred on average on day 8 of life (8.06 ± 6.053). In another retrospective study, Choreziak et al. (*n* = 163) found that thrombocytopenia prior to diagnosis but not immediately postpartum was associated with ROP (*p* = 0.015), with counts > 232 × 10^9^/L linked to spontaneous favorable outcomes, and lower mean values in the ROP group (325 vs. 401 × 10^9^/L; *p* = 0.008) [53].

This trend was also confirmed in our study. Thrombocytopenia was identified in 33.6% of preterm infants and was significantly more frequent in those with ROP (45.5%) compared with those without ROP (19.0%). Risk analysis indicated a 3.542-times increased risk of developing ROP in the presence of thrombocytopenia (*p* = 0.001; OR = 3.542; 95% CI: 1.636–7.668). In addition, platelet counts were consistently lower in the ROP group on days 7, 14, and 21 of life, with statistically significant differences observed on day 7 (212.95 ± 88.906 vs. 274.34 ± 115.747; *p* < 0.01).

This study has certain limitations that should be acknowledged. We did not assess the combined effect of anemia, thrombocytopenia, and transfusions on ROP risk. Given the variable hematological profiles of preterm infants in our cohort, such an analysis was not feasible. Instead, our focus was on dynamically monitoring these parameters at seven key time points during the first month of life to determine the moments at which they became statistically significant for ROP, as well as the onset of anemia and thrombocytopenia. Future larger cohorts will be required to explore these interactions in more depth. Through their ability to regulate proangiogenic factors such as VEGF, platelets directly influence the balance of retinal angiogenesis. Therefore, their involvement in retinal vascular development suggests that early management of thrombocytopenia could contribute to the prevention and control of ROP [50].

Recent literature has reported conflicting results regarding the role of platelet transfusions in the progression of ROP. On one hand, several observational studies have indicated a possible association between transfusion administration and an increased risk of ROP, as well as other systemic complications [47]. On the other hand, some clinical and experimental studies suggest that untreated thrombocytopenia may actively contribute to disease progression and that timely platelet transfusions could help limit pathological neovascularization [54].

Correction of thrombocytopenia has been associated, in some cases, with the spontaneous regression of Aggressive posterior retinopathy of prematurity (AP-ROP), suggesting a potential therapeutic benefit of this intervention [55]. In addition to clinical studies, experimental data also support the influence of platelet transfusions on the balance of retinal angiogenesis. In an oxygen-induced retinopathy mouse model, Cakir et al. showed that platelet administration during the active phase of neovascularization significantly reduced retinal vascular proliferation (−19.3%; *p* = 0.008). A notable reduction in VEGF-A expression was also observed, both at the mRNA level (*p* < 0.0001) and protein level (*p* = 0.011). This antiangiogenic effect was present only when platelets with intact granules were used, whereas activated and degranulated platelets did not affect the course of retinal angiogenesis [56].

In a cross-sectional study on over 1.78 million preterm infants with GA < 32 weeks or BW < 1500 g, Marwa et al. found ROP in 22.3% of those receiving platelet transfusions versus 19.2% without transfusions (*p* < 0.001) [57]. Similarly, a retrospective study from Portugal including 140 preterm infants with GA < 30 weeks identified platelet transfusions as an independent risk factor, with a more than fivefold increase in disease risk (OR = 5.28; 95% CI: 1.31–21.21; *p* = 0.019) [58]. In line with these findings, in our study, platelet transfusions were administered to 27 of the 140 preterm infants included (19.3%). The proportion was significantly higher among newborns with ROP (26.0%) compared with those without ROP (11.1%). Statistical analysis confirmed a significant association between platelet transfusion and the presence of ROP (Chi^2^ = 4.917, *p* = 0.027), with a 2.807-times increased risk of developing the disease in preterm infants who required transfusions (OR = 2.807; 95% CI: 1.100–7.160).

The results obtained suggest that platelet transfusion should not be interpreted as a causal factor for ROP, but rather as a potential indicator of the severity of the hematologic imbalance. Nevertheless, it remains essential for future research, particularly prospective and multicenter studies, to clarify the optimal timing, the appropriate transfusion profile, and the actual clinical impact of these interventions on the progression of ROP.

## 5. Conclusions

The results of our study provide a new perspective on the value of hematological parameters in anticipating the risk of ROP. By monitoring the longitudinal evolution of hemoglobin, RBC, platelets, and transfusion requirements during the first four weeks of life, significant differences were identified between preterm infants who developed ROP and those who did not. Notably, early trends in hematological dynamics revealed significantly lower RBC values in the ROP group on days 3 and 7, alongside reduced platelet counts on days 7, 14, and 21. Both anemia and thrombocytopenia were more prevalent among infants who developed ROP, being associated with over a threefold increase in disease risk. Furthermore, erythrocyte and platelet transfusions, more frequently administered in these patients, were associated with a significantly increased risk.

These findings highlight the relevance of these parameters as potential predictive markers capable of complementing current clinical criteria. Their integration into ROP screening strategies could contribute to a more accurate identification of high-risk preterm infants. Such an approach would allow for more efficient use of medical resources and support the development of a personalized care model focused on preventing visual complications. Correlating hematological values with transfusion history could serve as a valuable tool in neonatal practice.

In this context, there is a growing need for longitudinal studies to validate these markers and support their integration into clinical management protocols for ROP, based on a deeper understanding of neonatal hematologic instability.

## Figures and Tables

**Table 1:** Distribution of preterm infants by gestational age and presence of ROP.

		ROP Status				Pearson Chi-Squared Test
		ROP		Non-ROP		
		*n*	%	*n*	%	
Gestational age	<28 weeks	12	15.6%			Chi^2^ = 55.028
	28–30 weeks	37	48.1%	4	6.3%	*p* < 0.001
	31–32 weeks	20	26.0%	24	38.1%	
	33–34 weeks	8	10.4%	35	55.6%	
Total		77	100.0%	63	100.0%	

**Table 2:** Hemoglobin—analysis of values in the cohort and comparison between the ROP and non-ROP groups.

HGB	*n*	Mean	Standard Deviation	Min	Max	Median	IQR		t-Student/ Mann–Whitney/ANOVA/Kruskal–Wallis Test
							25th	75th	
TOTAL									
HGB 1	140	17.2094	4.13786	7.10	57.60	16.8000	15.4250	18.5000	
HGB 3	140	16.5614	3.42766	9.00	42.10	16.4500	14.4250	18.2750	
HGB 5	140	15.7271	2.54148	8.30	22.10	15.6000	14.0000	17.6750	
HGB 7	140	14.6964	2.55538	9.50	20.80	14.8000	12.6000	16.6750	
HGB 14	131	13.5031	3.67421	7.80	44.70	13.3000	11.7000	14.9000	
HGB 21	124	12.0242	3.12663	7.70	35.00	11.6000	10.1250	13.1000	
HGB 28	111	10.7946	2.77842	1.60	28.30	10.5000	9.2000	12.0000	
HGB 1									
ROP	77	17.4625	5.23090	7.10	57.60	16.8000	15.7000	18.6000	U = 2309.500
non-ROP	63	16.9000	2.16065	13.00	23.30	16.9000	15.2000	18.5000	*p* = 0.627
HGB 3									
ROP	77	16.4182	3.99818	9.00	42.10	15.9000	14.4000	17.8000	U = 2038.500
non-ROP	63	16.7365	2.58621	11.70	21.30	17.0000	15.3000	18.5000	*p* = 0.105
HGB 5									
ROP	77	15.4506	2.67467	8.30	22.10	15.2000	13.8500	16.9500	t = −1.428
non-ROP	63	16.0651	2.34536	11.70	20.10	16.5000	14.1000	18.0000	*p* = 0.155
HGB 7									
ROP	77	14.3104	2.68179	9.50	20.80	14.3000	12.2500	15.8000	t = −1.997
non-ROP	63	15.1683	2.32648	10.80	19.80	15.2000	13.3000	16.9000	*p* = 0.048
HGB 14									
ROP	77	13.3506	4.43861	7.80	44.70	12.8000	11.0000	14.4500	U = 1674.000
non-ROP	54	13.7204	2.18635	9.40	18.60	13.4500	12.1500	15.2000	*p* = 0.058
HGB 21									
ROP	75	11.8893	3.64241	7.70	35.00	11.6000	9.3000	13.1000	U = 1560.500
non-ROP	49	12.2306	2.12722	8.30	17.40	11.7000	10.9000	13.2000	*p* = 0.157
HGB 28									
ROP	74	10.8649	3.12583	1.60	28.30	10.5500	9.3000	12.0000	U = 1302.500
non-ROP	37	10.6541	1.93500	7.90	15.40	10.2000	9.2000	12.0000	*p* = 0.677

**Table 3:** Red blood cells—analysis of values in the cohort and comparison between the ROP and non-ROP groups.

RBC	*n*	Mean	Standard Deviation	Min	Max	Median	IQR		t-Student/Mann–Whitney/ANOVA/Kruskal–Wallis Test
							25th	75th	
TOTAL									
RBC 1	140	4.5078	0.59300	1.98	6.17	4.4800	4.1075	4.8975	
RBC 3	140	4.4043	0.71631	2.38	6.42	4.4200	3.8475	4.8500	
RBC 5	140	4.3024	0.67117	2.79	5.82	4.3050	3.8700	4.7900	
RBC 7	140	4.0536	0.67613	2.46	5.71	4.0500	3.5625	4.5675	
RBC 14	131	3.8711	1.03540	2.22	12.80	3.7600	3.4100	4.2500	
RBC 21	125	3.5480	1.00112	2.14	11.80	3.4600	3.0000	3.9000	
RBC 28	109	3.2355	0.62008	1.79	5.00	3.1100	2.7850	3.6600	
RBC 1									
ROP	77	4.4566	0.60374	1.98	5.58	4.4700	4.0000	4.8700	t = −1.130
non-ROP	63	4.5703	0.57822	3.26	6.17	4.5600	4.2300	4.9000	*p* = 0.261
RBC 3									
ROP	77	4.2675	0.72217	2.38	6.42	4.2700	3.7800	4.6000	t = −2.546
non-ROP	63	4.5714	0.67781	3.14	5.81	4.5900	4.1000	5.1000	*p* = 0.012
RBC 5									
ROP	77	4.2119	0.68960	2.79	5.82	4.2600	3.8350	4.5900	t = −1.777
non-ROP	63	4.4130	0.63591	3.29	5.54	4.4800	3.8700	4.8900	*p* = 0.078
RBC 7									
ROP	77	3.9303	0.69662	2.46	5.71	3.9500	3.5050	4.3650	t = −2.429
non-ROP	63	4.2044	0.62292	2.98	5.54	4.2200	3.6900	4.7200	*p* = 0.016
RBC 14									
ROP	77	3.8339	1.25248	2.22	12.80	3.7300	3.3300	4.2100	U = 1740.000
non-ROP	54	3.9241	0.61257	2.70	5.56	3.8750	3.5425	4.4225	*p* = 0.113
RBC 21									
ROP	75	3.5216	1.19604	2.14	11.80	3.4000	2.8300	3.9100	U = 1588.000
non-ROP	50	3.5876	0.61096	2.45	5.15	3.5000	3.2625	3.7950	*p* = 0.148
RBC 28									
ROP	72	3.2413	0.63456	1.79	5.00	3.1050	2.8050	3.7500	U = 2001.000
non-ROP	37	3.2243	0.59931	2.31	4.47	3.2300	2.7800	3.4800	*p* = 0.828

**Table 4:** Distribution of preterm infants by anemia and ROP status.

Anemia		ROP Status				Total	
		ROP		Non-ROP			
		*n*	%	*n*	%	*n*	%
	present	64	83.1%	38	60.3%	102	72.9%
	absent	13	16.9%	25	39.7%	38	27.1%
		Pearson Chi-squared test: Chi^2^ = 9.108/*p* = 0.003 OR = 3.239/95%CI = (1.482 ÷ 7.074)					
Total		77	100.0%	63	100.0%	140	100.0%

**Table 5:** Day of anemia onset—comparative analysis by ROP status.

Day of Anemia Onset	*n*	Mean	Standard Deviation	Min	Max	Median	IQR		Mann–Whitney/Kruskal–Wallis Test
							25th	75th	
TOTAL	102	13.59	7.606	1	28	12.50	6.00	21.00	
ROP status									U = 1083.500
ROP	64	13.03	7.307	1	28	12.00	6.00	19.75	*p* = 0.358
non-ROP	38	14.53	8.097	2	28	15.00	6.75	21.25	

**Table 6:** Distribution of preterm infants according to RBC transfusion and ROP status.

Red Blood Cell Transfusion		ROP Status				Total	
		ROP		Non-ROP			
		*n*	%	*n*	%	*n*	%
	Present	44	57.1%	19	30.2%	63	45.0%
	Absent	33	42.9%	44	69.8%	77	55.0%
		Pearson Chi-squared test: Chi^2^ = 10.194/*p* = 0.001 OR = 3.088/95%CI = (1.530 ÷ 6.232)					
Total		77	100.0%	63	100.0%	140	100.0%

**Table 7:** Platelet count—analysis of values in the cohort and comparison between the ROP and non-ROP groups.

PLT	*n*	Mean	Standard Deviation	Min	Max	Median	IQR		t-Student/Mann–Whitney/ANOVA/Kruskal–Wallis Test
							25th	75th	
TOTAL									
PLT 1	140	232.16	75.325	28	479	229.50	180.75	285.25	
PLT 3	140	207.11	78.258	21	430	199.50	157.00	256.25	
PLT 5	140	209.03	80.006	35	416	206.00	155.25	258.50	
PLT 7	139	240.33	105.892	53	721	228.00	168.00	297.00	
PLT 14	130	274.07	132.394	47	683	264.00	167.50	373.50	
PLT 21	124	308.02	156.271	40	718	300.00	170.00	429.00	
PLT 28	111	296.59	156.638	45	847	287.00	168.00	416.00	
PLT 1									
ROP	77	229.73	79.086	28	479	228.00	177.00	279.00	t = −0.422
non-ROP	63	235.14	70.972	42	414	233.00	180.00	286.00	*p* = 0.674
PLT 3									
ROP	77	199.16	84.140	21	430	188.00	149.00	252.00	t = −1.333
non-ROP	63	216.83	69.842	32	416	221.00	169.00	261.00	*p* = 0.185
PLT 5									
ROP	77	199.08	76.093	35	416	201.00	151.50	248.50	t = −1.637
non-ROP	63	221.19	83.544	35	394	221.00	168.00	277.00	*p* = 0.104
PLT 7									
ROP	77	212.95	88.906	53	420	202.00	160.50	261.50	t = −3.536
non-ROP	62	274.34	115.747	61	721	262.50	191.75	346.00	*p* = 0.001
PLT 14									
ROP	76	237.42	122.846	47	549	226.00	133.75	312.00	t = −3.951
non-ROP	54	325.65	129.117	70	683	347.50	230.00	394.25	*p* < 0.001
PLT 21									
ROP	75	276.37	161.710	40	622	256.00	135.00	405.00	t = −2.871
non-ROP	49	356.47	135.286	112	718	358.00	271.50	442.00	*p* = 0.005
PLT 28									
ROP	74	281.34	161.911	45	847	288.00	143.00	398.00	t = −1.458
non-ROP	37	327.08	142.749	111	607	269.00	212.00	449.50	*p* = 0.148

**Table 8:** Distribution of preterm infants by thrombocytopenia and ROP status.

Thrombocytopenia		ROP Status				Total	
		ROP		Non-ROP			
		*n*	%	*n*	%	*n*	%
	present	35	45.5%	12	19.0%	47	33.6%
	absent	42	54.5%	51	81.0%	93	66.4%
		Pearson Chi-squared test: Chi^2^ = 10.835/*p* = 0.001 OR = 3.542/95% CI = (1.636 ÷ 7.668)					
Total		77	100.0%	63	100.0%	140	100.0%

**Table 9:** Day of thrombocytopenia onset—comparative analysis by ROP status.

Day of Thrombocytopenia Onset	*n*	Mean	Standard Deviation	Min	Max	Median	IQR		Mann–Whitney/Kruskal–Wallis Test
							25th	75th	
TOTAL	47	7.49	5.830	1	21	5.00	3.00	14.00	
ROP status									U = 160.000
ROP	35	8.06	6.053	1	21	6.00	3.00	14.00	*p* = 0.220
non-ROP	12	5.83	4.988	1	14	4.50	2.00	11.25	

**Table 10:** Distribution of preterm infants according to platelet transfusion and ROP status.

Platelet Transfusion		ROP Status				Total	
		ROP		Non-ROP			
		*n*	%	*n*	%	*n*	%
	present	20	26.0%	7	11.1%	27	19.3%
	absent	57	74.0%	56	88.9%	113	80.7%
		Pearson Chi-squared test: Chi^2^ = 4.917/*p* = 0.027 OR = 2.807/95% CI = (1.100 ÷ 7.160)					
Total		77	100.0%	63	100.0%	140	100.0%

## Data Availability

No new data were created or analyzed in this study. Data sharing is not applicable to this article.

## Abbreviations

The following abbreviations are used in this manuscript:

ROP	Retinopathy of prematurity
PMA	Postmenstrual age
VEGF	Vascular Endothelial Growth Factor
HIFs	Hypoxia-inducible factors
IGF-1	Insulin-like Growth Factor 1
RBC	Red blood cell
NICU	Neonatal intensive care unit
HbF	Fetal hemoglobin
HbA	Adult hemoglobin
GA	Gestational age
BW	Birth weight
Hb	Hemoglobin
PLT	Platelet
AP-ROP	Aggressive posterior retinopathy of prematurity
